# Composition and Hierarchical Organisation of a Spider Silk

**DOI:** 10.1371/journal.pone.0000998

**Published:** 2007-10-03

**Authors:** Alexander Sponner, Wolfram Vater, Shamci Monajembashi, Eberhard Unger, Frank Grosse, Klaus Weisshart

**Affiliations:** 1 Department of Zoology, University of Oxford, Oxford, United Kingdom; 2 Leibniz Institute for Age Research, Fritz Lipmann Institute, Jena, Germany; Technische Universität München, Germany

## Abstract

Albeit silks are fairly well understood on a molecular level, their hierarchical organisation and the full complexity of constituents in the spun fibre remain poorly defined. Here we link morphological defined structural elements in dragline silk of *Nephila clavipes* to their biochemical composition and physicochemical properties. Five layers of different make-ups could be distinguished. Of these only the two core layers contained the known silk proteins, but all can vitally contribute to the mechanical performance or properties of the silk fibre. Understanding the composite nature of silk and its supra-molecular organisation will open avenues in the production of high performance fibres based on artificially spun silk material.

## Introduction

Spider silks display extraordinary mechanical features [1] and the toughness of some dragline or major ampullate silks even rival man-made high-tech fibres [2]. The major constituents of silks are proteins termed spidroins, which are synthesized in specialized glands and are of high molecular weight ranging 200–350 kDa in size [3]. Orb weaving spiders can produce up to seven different silk types and their fibres are composed of one or two spidroins, all of which are encoded by members of one gene family [4]. Spidroins show a modular structure consisting of a long repetitive sequence that is flanked by non-repetitive conserved N- and C-terminal regions [4], [5]. Repetitions are formed by tandemly arranged sequence blocks ∼150–500 amino acids in length that either represent themselves the basic repetition units or that can be subdivided into shorter ensemble repeats and repetitive amino acid motifs [6]–[8].

The properties of dragline silk are believed to depend equally on both the molecular design of its two main protein components termed major ampullate spidroins 1 (MaSp1) and 2 (MaSp2) and the hierarchical organisation of structural elements [9]–[12]. Both major ampullate spidroins are rich in glycine and alanine residues which form short GGX, GA and GPGXX (X, subset of amino acids) as well as poly-A motifs [6]. Poly-A and GA sequences organize into stacked anti-parallel β-pleated sheets that form crystallites, which are essentially randomly oriented [13], [14]. The amide to amide interactions of the protein backbones in the β-sheets are thought to give a strong molecular cohesion and thus confer strength to the fibre. These crystalline areas are embedded in an amorphous matrix that is formed by the glycine enriched motifs (GGX in MaSp 1, GPGXX in MaSp 2), which adopt 3_1_-helical and type II β-turn structures [15]. These amorphous regions are believed to confer elasticity to the fibre [3], [15].

Based on morphological evidence a hierarchical organisation of the fibre into distinct structural elements has been reported, in particular an inner core was discriminated from an outer skin layer and fibrils running parallel to the fibre axis have been observed [11], [16], [17]. However, the bona fide existence and the total number of different structural elements as well as their origin are still contentious issues [9]–[11]. As long as biochemical data on such structures are missing models based on these must remain vague.

Due to silk's biomimetic potential attempts are ongoing to mass produce parts of the known repetitive sequences of spidroins and to spin the material into fibres [18]–[21]. However, the native properties of silk have not been matched yet. This might in part be due to the fact that no full length sequences have been expressed, since N- and C-termini might contribute to fibre formation [6]. On the other hand, the spinning process is believed to also vitally contribute to the properties of native spider silk and influence its supra-molecular structure [22]. For example, the major ampullate silk's properties vary significantly in dependence of the spinning speed [23], [24]. For producing the dragline this type of silk is fast spun, resulting in a stiff fibre that can support the body weight of the spider. In contrast, if the silk is used as the frame thread of the orb-web, it is spun with an order of magnitude slower speed and displays more extensibility, which is needed to dissipate the energy of prey impact on the web [25]. In order to design a successful spinning strategy a profound knowledge of the composition and structure of the silk fibres in addition to the complete primary structure of their protein constituents seems therefore imperative. Here we report on the biochemical nature of distinct structural elements and integrate previous reported morphological data into a revised model of silk structure and its properties.

## Results

### Morphological appearance of dragline silk

**Figure 1:**
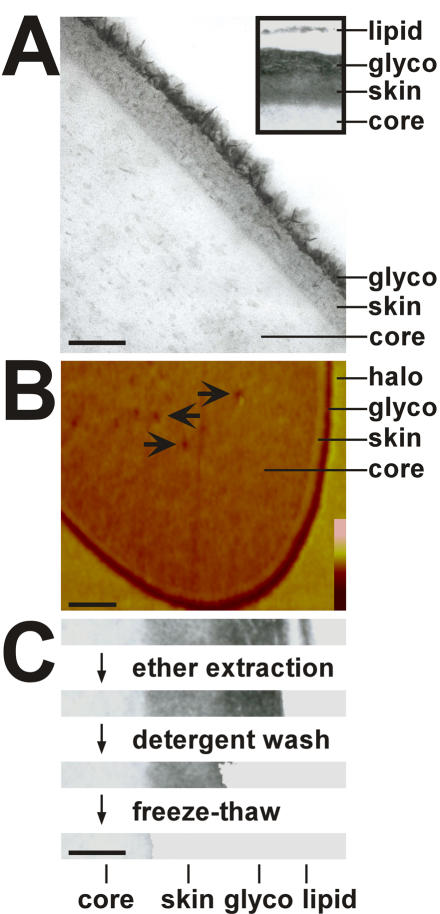
Cross sections of dragline from the spider *Nephila clavipes*. Panel A. TEM micrographs. The core, skin and glyco layers are indicated. The inset shows the presence of a lipid layer detached from the rest of the fibre. Bar corresponds to 100 nm. Panel B. AFM micrographs. The arrows indicate some of the cavities. The core, skin, glyco layers and an artificial halo are indicated. Lack of symmetry in the latter indicates that the halo is caused by the sectioning procedure and reflects material compressed and displaced by the knife as it approaches the hard skin layer. The bar corresponds to 500 nm. The height colour coding bar extends bottom to top from 0 to 100 nm. Panel C. Attachment strength of silk layers. Untreated fibres were successively ether extracted, vigorously washed in 0.1% Triton X-100 detergent solution and subjected to freeze-thaw cycles. After treatment cross-sections of fibres for each step were analyzed by TEM. The loss of layers from outside to inside is evident. The efficiencies for removal lipids, glycoproteins and the skin were 100%, 70% and 30%, respectively. The bar corresponds to 200 nm.

Transmission Electron (TEM) and Atomic Force Microscopy (AFM) micrographs of cross-sectioned dragline silk fibres from the spider *Nephila clavipes* confirmed a core-skin structure, but revealed in addition two thin layers on top of the skin (Figure 1A and B). The exterior of these two layers is a membrane like material with a thickness of 10–20 nm (Figure 1A, inset), which was detached from the next layer or lost in most cases during the sectioning procedure. It was only loosely associated with the inward material since it could be removed by a gentle wash in phosphate buffered solutions (data not shown) or by ether extraction (Figure 1C).

The more interior of the outer two layers exhibited a thickness of approximately 40–100 nm and was distinguished in TEM images by its strong osmiophilic nature that is a strong staining with osmium tetroxide (Figure 1A). The layer consisted of fine fibrils embedded in an amorphous matrix. It was attached more tightly to the adjacent inner material, since it could be removed only by vigorous washing in detergent solutions (Figure 1C). In AFM the layer was deepened, which speaks for the softness of that material (Figure 1B).

Below the inward of the outer two tiers a skin layer of 50–100 nm was discernible in TEM, which was richer in contrast than the subjacent core material (Figure 1A). This layer was tightly attached to the inwards material, since it could be removed only by flash freeze-thaw cycles and hence by high stress to the fibre (Figure 1C). In AFM the material of this layer appeared elevated above the level of the section surface hinting to a more rigid structure of this part (Figure 1B). The elevation was caused by the knife during ultra sectioning at sites where it encountered more rigid material and higher forces.

The core layer comprises most of the fibre. Speckles of higher contrast are visible throughout the core's extension in TEM images (Figure 1A). In AFM numerous cavities were visible (Fig. 1B). The material was less elevated than the skin and hence of a softer nature.

### Biochemical composition of structural elements

**Figure 2:**
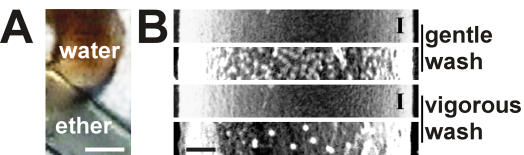
Biochemical composition of outer layers. Panel A. Oil red staining of fibres. Silk fibres were treated with water (upper filament) or ether extracted (lower filament) followed by oil red staining. Bar corresponds to 2000 nm. Panel B. Concavalin A (Con A) staining of fibres. Fibres gently washed in phosphate buffered saline or vigorously washed in 0.1% Triton X-100 were reacted with biotinylated Con A. The presence of α-methyl mannoside (α-MM), an inhibitor of Con A, is indicated by “I”. Bound Con A was visualised by gold conjugated streptavidin and SEM. The bar equals 500 nm.

**Figure 3:**
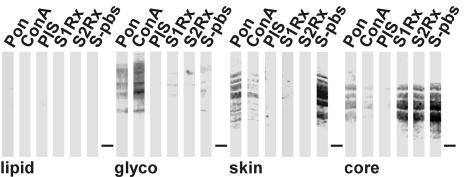
Protein composition of silk layers. Filter strips obtained by western blotting and loaded with material extracted from the indicated fibre layers were stained with Ponceau S (Pon) or reacted with Concavalin A (ConA), pre-immune serum (PIS), S1Rx, S2Rx and S-pbs. The running position of a 200 kDa marker is indicated by the lines.

A lipid character of the outermost layer was indicated by the weak staining of silk with the lipophilic dye oil red (Figure 2A). This staining was lost by ether extraction coincident with a loss in the layer (Figure 1C). The material did not show any prominent protein bands in western blots nor was the material reactive to Concavalin A or any of our employed silk specific sera, which included reactivity against the repetitive parts of MaSp1 (S1Rx) and MaSp 2 (S2Rx) and native silk (S-pbs) [26] (Figure 3). The layer therefore is likely to consist of previously described lipids [27] and we refer to it as the lipid coat.

Glycosylation that has been previously demonstrated for dragline silk [28] was in the majority associated with the next inward layer, since its removal by vigorous washing lead to a great reduction of Concavalin A reactivity on the surface of the fibre (Figure 2B). In addition the material of this layer was most strongly reactive to Concavalin A in western blots (Figure 3). The proteins extracted from that layer showed sizes above 200 kDa similar to the known spidroins, but reacted with none of the silk specific sera. Due to its high glycoprotein content we refer to this layer as the glyco coat.

The material of the next inward skin layer is composed of proteins that mostly displayed molecular weights similar to the known MaSps, but that were immunological distinct to them (Figure 3). They reacted exclusively with sera derived against native silk (S-pbs), but not with those specific for MaSp 1 (S1Rx) and 2 (S2Rx). The material was also reactive to Concavalin A, albeit to a lesser extent than the glycoprotein layer. Interestingly, two of the protein bands slightly exceeded the sizes of the known major ampullate spidroins.

The inner material is represented by the core and is made up by the known spidroins since it reacted with all silk specific sera (Figure 3). The core material reacted in addition with Concavalin A, but like the skin to a weaker extent than the glycoprotein layer.

### Distribution of different components

**Figure 4:**
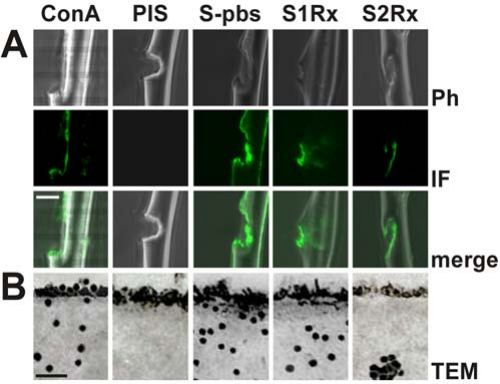
Localization of components. Panel A. Fluorescence labelling of silk fibres. Reactivities of Concavalin A (ConA), pre-immune serum (PIS), S-pbs, S1Rx and S2Rx to lesions of vigorously washed silk filaments. Phase contrast (Ph), immunfluorescence (IF) and merged images are shown. The bar indicates 4000 nm. Panel B. Immunogold staining of silk fibre cross-sections. Shown are transmission electron microscopy (TEM) images of dragline cross sections reacted with Concavalin A (ConA) or the indicated antibodies. The bar equals 200 nm.

The restriction of the two known MaSps to the core was proved by staining laser ablated fibres and fibre cross-sections with silk specific antibodies. Antibodies specific for MaSp 1 (S1Rx) and MaSp 2 (S2Rx) reacted exclusively with the inner material at lesions, but not the surface of the fibre (Figure 4A). In contrast, antibodies specific for native silk (S-pbs) were reactive to both sites. Specificity of the reaction was provided by pre-immune serum (PIS) that was negative in either case. In TEM cross-sections a subdivision of the core into an outer and inner layer with extensions of 300–400 nm and 1800–2300 nm, respectively, based on the distribution of the two known spidroins was evident. MaSp1 was homogeneously located throughout the core, whereas MaSp2 was found exclusively within the inner region in a more clustered staining pattern in accordance to previous findings (Figure 4B) [29]. MaSp 1 and 2 epitopes were absent from the skin, suggesting their absence from this layer. Although we cannot rule out epitope masking by chemical modification or differential polymerisation, we want to point out that the skin material was neither reactive to silk sera specific for the conserved C-termini of the MaSps [30] nor to any of the other MaSp specific sera after its solubilisation (Figure 3). This strongly suggests that the skin is not formed by the two known spidroins. Proteins differed also between the skin and the glyco layer since antibodies directed against native silk did not react with the latter, but exclusively with the skin and the core.

Glycosylation was evident in the glyco and skin layers as well a throughout the core. Reactivity of Concavalin A per area was strongest for the glyco layer, followed by the skin and the core in accordance to their reactivity in western blots (Figure 3). This indicates different degrees of glycosylation of the different layers.

### Physicochemical properties of layers

**Figure 5:**
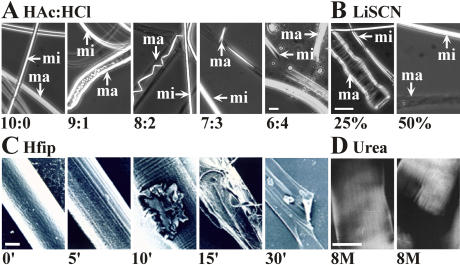
Treatment of fibres with chaotropic agents and acids. Panel A. Light Microscopy (LM) phase images of fibres treated with mixtures of different ratios between HAc and HCl. Bar corresponds to 4000 nm. Minor (mi, reinforcement) and major (ma, dragline) ampullate fibres are indicated. Panel B. Light Microscopy (LM) phase contrast images of dragline treated with LiSCN at the stated concentrations for 1 minute. Bar corresponds to 8000 nm. Panel C. Scanning Electron Microscope (SEM) images of dragline incubated for the indicated times with 99% Hfip. Bar corresponds to 1000 nm. Panel D. Video contrast images of draglines (left unstressed, right fractured by applying stress) soaked in 8 M urea and counterstained with Coomassie Brilliant Blue. Bar equals 4000 nm.

Evidence for a robust nature of the skin was found in experiments where filaments were treated with chaotropic agents or acids. In both cases the skin showed increased resistance to these chemicals compared to the core (Figure 5A and B). In acids, in dependence of their strength, a two step dissolution process of a dragline was observed (Figure 5A). In the first step (HAc:HCl ratios down to 8∶2) the core material condensed to light diffracting filaments that detached from the outer skin and kinked. Accompanying to filament detachment was the expansion of the fibre diameter. In the second phase using higher HCl concentrations the inner filaments were dissolved and the outer skin ruptured. These processes hinted to a substantial pressure built-up inside the fibre. The skin deformed plastically since even in cases, where the core material dissolved and depleted from the inner volume as was the case with lithium thiocyanate (LiSCN), it did not contract to its original size (Figure 5B). Interestingly, the dissolution behaviour of minor ampullate silk was very similar to that of the skin. It seemed to be uninfluenced at low and dissolved only at high concentrations of acids and chaotropic solutions (Figure 5).

If treated with hexafluorisopropanol (Hfip) grooves on the surface became obvious that first ran parallel and at later time points of treatment also perpendicular to the fibre axis (Figure 5C). At restricted sites where Hfip was able to attack and peel of the skin, it lead to dissolution of the inner material with intermediate fibrillar like structures. The skin like remnants of the dissolution process did not display similar patterns.

**Figure 6:**
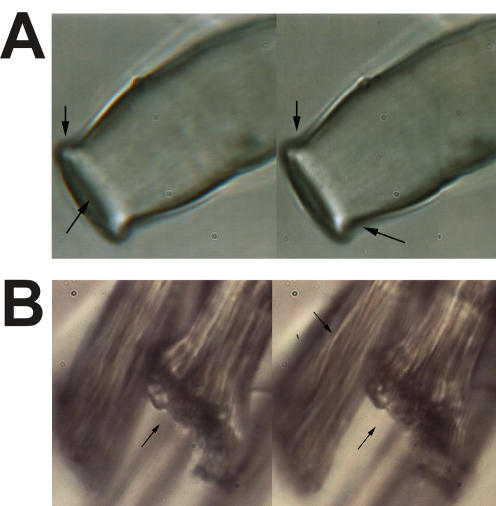
Spatial image microscopy of fibrillar structures. Panel A. Cut end of a dragline incubated in 8 M urea. High resolution stereo image was acquired with a Zeiss Neofluar 100x/NA 1.3 oil immersion objective. A stereo lorgnette is required for obtaining the 3D effect. The upper arrows will appear as one arrow behind the object, whereas the lower arrow in the right picture will appear in front of it. They indicate the end of the conical shape pointing to the open ends. Fibrils can be seen in the central parts (lower arrow, left image) of the fibre. Panel B. Freeze fractured dragline incubated in 8 M urea. Image was acquired as in panel A. The fractured site is indicated by the lower arrows. The exposed interior shows that the fibre consists of many fibrils, which are twisted (upper arrow) and from bundles.

Urea treatment induced swelling of the fibre resulting in diameters of ∼8 µm without dissolving the material (Figure 5D). Obviously, the core material expanded against the resistance of the skin. Further evidence for this assumption was provided by the observed slight conical forms of the cut ends of fibres soaked in 8 M urea (Figure 6A). The ends were tapered since the pressure was less strong at these sites than within the fibre [31].

### Structural organisation of the core

Particularly by video contrast microscopy (VCM) of urea treated and Coomassie stained samples fibrils were evident, which were oriented parallel to the fibre axis (Figure 5D). These fibrils could be noticed most pronounced at the fractured end of fibres. The resolution of spatial image microscopy attributed these fibrils to the inner of the expanded filament (Figure 6A). Bundling of the fibrils became especially obvious at the ends of fractured silk (Figure 6B). Fibrils within the bundles appeared twisted.

### Solubilisation of MaSp 1 and 2 components within the fibre

**Figure 7:**
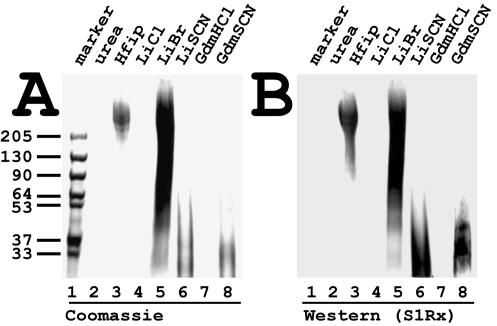
Solubilisation of polymerised spidroins. Dragline silk from *N. clavipes* was incubated with the indicated solvents and the soluble fraction was analysed by gel-electrophoresis followed by Coomassie staining (A) and western blotting employing antibodies S1Rx and enhanced luminescence (ECL) for detection. The molecular weights of marker proteins are indicated in kilo Daltons (kDa).

If dragline was incubated in urea or chloride salts of lithium (LiCl) and guanidinium (GdmCl) only a swelling of the fibre was noticed (Figure 5D for urea; data not shown for LiCl and GdmCl). There was no noticeable protein quantity solubilised in these cases (Figure 7A lanes 2, 4 and 7). In contrast hexafluorispropananol (Hfip) and lithium bromide (LiBr) extracted high molecular weight material (Figure 7A lanes 3 and 5). The material reacted with silk specific sera (shown for S1Rx in Figure 7B; data not shown for S2Rx and S-pbs) identifying the material as major ampullate spidroins. For both solvents, fragmentation of the high molecular weight material became only obvious after extended incubation (>24 hrs) as exemplified in Figure 7. No fragmentation was obvious for shorter incubation periods in accordance to previous results [26]. In contrast treatment with thiocyanate salts of lithium (LiSCN) and guanidinium (GudmSCN) lead to a rapid fragmentation as demonstrated previously for LiSCN (data not shown) [26]. Dependent on the length of incubation fragments became increasingly smaller (data not shown). Long incubation times eventually resulted in a complete break-down of the high molecular weight material into small peptides below 30 kDa in size (Figure 7A lanes 6 and 8). The silk specific antibodies identified these peptides as MaSp 1 and 2 derivates (Figure 7B).

## Discussion

### Hierarchical structure of silk

Whereas the dragline fibre of *Nephila clavipes* looked unstructured in transmission electron microscopy (TEM), the dragline of *N. edulis* showed the existence of a multilayer assembly [10], [11], [32]. Based on additional evidence by atomic force microscopy (AFM) and video contrast microscopy (VCM) a model was proposed that divides the dragline thread or filament into a core and a skin [17], [31]. We provide strong and independent evidence for such a core-skin structure for *N. clavipes*. First, the skin could be mechanically separated from the core by freeze-thaw cycles (Figure 1) [33]. Second, the skin showed increased resistance to chaotropic agents and acids compared to the core (Figure 5). And third, the skin was biochemically distinct from the core (Figure 2). We also provide a biochemical basis for the subdivision of the core into an outer and inner region [17]. The confinement of MaSp 2 to the inner part of the core matched in its extension an area of decreased β-sheet content [34] (Figure 4). Grooves that became evident on the surface of the fibre after treatment with hexafluorisopropanol might match an interwoven fibrillar network reported for the skin (Figure 5C) [31]. However, since the skin like remnants of the dissolution process did not exhibit such structures, we believe them to be the result of an imprint of the inner core material onto the outer skin and rather stay in line with studies reporting a uniform skin surface [17], [35], [36]. We attribute fibrils running parallel to the fibre axis to the core area based on spatial image microscopy (Figure 6A). The optical resolution of this technique was however not sufficient to estimate the diameters of the fibrils. We believe that they correspond to fibrils seen in the core of the draglines of various spiders that varied in diameter between 100 and 150 nm and the silk of the moth *Bombyx mori* [16], [17], [28], [37]. Interestingly, fibrils were twisted (Figure 6B). Similar to a twisted cable rope, crack propagation will be minimized with such an arrangement [28], [36].

The existence of bona fide fibrils has been, however, questioned since the dragline did not display a typical kinking that should be observed if material consisting of highly oriented fibrils is compressed [9]. Such a lack in kinking could, however, be easily explained by the presence of the skin and its plasticity. Thus, fibrils seemed not purely the artificial result of longitudinal fractures in the highly oriented thread material, caused for example by breaks and ruptures in the silk material, but rather a consequence of the inbuilt material heterogeneities.

### Composition of layers and their contribution to silk performance

**Figure 8:**
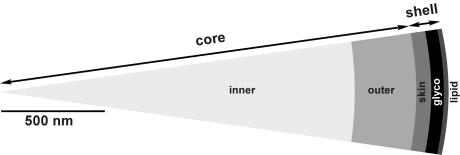
Model of the multilayer organisation of dragline silk. A dragline can be divided into a shell and a core with five major layers of different material composition (from exterior to interior): a lipid coat, a glyco coat, a skin, an outer core and inner core. The approximate relative extensions of each layer are indicated.

The complexity in the morphology of spider dragline silk of *N. clavipes* was mirrored in an equally complex composition of the underlying structures (Figures 1, 2 and 3). Each of the five different layers possessed some unique compounds and performed different functions within the silk fibre (Figure 8 and Table 1). We have divided the fibre into a shell and a core. The shell comprises a lipid coat, a glyco coat and the skin. The core is bipartite and can be subdivided into an inner and outer region.

**Table 1:** Compositions and functions of silk layers from the dragline of *N. clavipes*

layer	radial dimension (nm)	composition	function
lipid coat	10–20	lipids	protection
			social aspects
glyco coat	40–100	glycoproteins related to spidroins	protection
			pliancy
			water balance
skin	50–100	MiSp-like proteins	plasticity
			support
outer core	300–400	MaSp 1	tensile strength
			rigidity
inner core	1800–2300	MaSp 1 and 2	tensile strength
			elasticity

MiSp: minor ampullate spidroin; MaSp: major ampullate spidroin

The outermost layer is the lipid coat. The absence of proteins and its staining with a lipophilic dye suggests that this layer is exclusively formed by lipids (Figures 2 and 3). It is most likely identical to a membrane like lipid layer in *N. edulis* [11]. Lipids may provide some protection against environmental impact and micro-organisms but their main function is to serve as carriers for pheromones in sex and species recognition [27]. Its loose attachment suggests that the lipid coat will not substantially contribute to the mechanical performance of the fibre (Figure 1).

Due to its tighter attachment than the lipid layer the glyco layer seems more likely to function as the interface of the fibre to its environment and may provide protection against microbial attack. In addition, due to its high content in glycoproteins (Figure 2), this layer could be indirectly important for the mechanical strength in regulating the water balance, which has an impact on the contraction state of the fibre [38]. It may also act as a lubricant to add pliancy to the fibre. The proteins of the glyco layer are different to those from the skin and core, albeit their molecular weights are comparable suggesting a higher complexity in the protein components of a silk fibre than presently anticipated (Figure 3).

The high resistance of the skin to chaotropic agents and acids argues for a dual role of this layer (Figure 5). First, it provides protection against environmental damage by chemical agents and microbial activities. Second, it represents a workable structure that adds plasticity to the fibre and acts as a mechanical support confining the core material. The skin contained silk proteins of equal and higher molecular weight than the known spidroins (Figure 3). A certain length restriction might therefore be required for all silk proteins for efficient alignment and aggregation [6]. Skin proteins were glycosylated to a lesser extent than the silk proteins from the glyco layer. The sugar components nevertheless may affect the alignment and aggregation behaviour of the spidroins in dependence of the pH environment in the duct [26]. Since the chemical resistance of the skin was similar to the minor ampullate filaments putative skin spidroins might bear primary structure designs similar to minor ampullate spidroins (MiSp) [39]. The skin could be separated from the inward material only by harsh treatments, preferentially flash freeze-thaw cycles, suggesting a tight association with the core proteins.

The core contained the two known spidroins. The outer core area was shown previously to possess increased β-sheet content and it was suggested to have a different protein composition than the core [17], [34]. Indeed the outer core was devoid of MaSp 2 (Figure 4), which due to its proline content was predicted to impede in the formation of β-sheet structures, to reduce the lateral growth of crystallite areas, and to be excluded from areas of high β-sheet content [10], [23], [29]. The inner core showed the presence of cavities termed canaliculi in AFM (Figure 1B) and TEM [28], [36]. Their spherical precursor droplets and MaSp 2 were argued to represent weak points in the inner core that render the material more prone to fibril formation when a load is applied [22], [29]. If fibrils are built already during the formation of the fibre as it is actively drawn by the spider or in later use is currently unknown. The predicted properties of the two spidroins [3], [10], [23] and molecular models of dragline silk [15], [40], [41] suggest at any rate that the outer core as well as the fibrils should have a higher degree of molecular orientation with higher coherence and hence should be more rigid, whereas the MaSp 2 rich interspaces between the fibrils should form a less well organized and more flexible matrix [29].

MaSp 1, however, seemed to be missing in the draglines produced by spiders of the genera *Araneus*. Instead two types of MaSp 2 proteins (MaSp 2a and MaSp 2b, also designated ADF-3 and ADF-4 in *A. diadematus*) have been identified [42], which up to now presents a unique situation for araneoid spiders [43]. Nevertheless, albeit both MaSp2s are rich in proline, their aggregation behaviour and hydropathicity characteristics are quite distinct [43], [44]. ADF-3 is more hydrophilic and remained soluble if expressed in insect cells, whereas ADF-4 sequences readily aggregated and formed filaments. In this respect, ADF-4 and ADF-3 strongly resemble MaSp 1 and MaSp 2, respectively, in their biophysical characteristics [43]. Proline containing proteins do not abolish but only hinder in β-sheet formation. They will eventually be incorporated in β-crystals in the absence of non-proline containing spidroins, although the overall energy costs will be higher to stretch the molecules for perfect parallel alignment. We would therefore predict a similar polymerisation mechanism of MaSp 2b and 2a compared to the one discussed for MaSp 1 and 2 and as a result a likeness in the structural organisation of the resulting fibre. This is supported by the nearly identical mechanical performances and dissolution characteristics against chaotropic agents of the different dragline types (Figure 7) [1], [43].

### Implications for fibre spinning

The protein feedstock for dragline silk is produced and stored in the major ampullate gland [22]. The gland shows regionalization and can be divided into an A-, intermediate and B-zone, the latter leading over to the duct [45]. B-zone extracts contained some silk proteins that were higher in molecular weight than those isolated from the A-zone [26]. Their sizes and reactivity to silk specific sera matched proteins extracted from the skin (Figure 3). We therefore want to suggest that in the A-zone the bulk of the spinning dope consisting of MaSp1 and 2 is produced, which will compose the core of the fibre, while the B-zone secretes the skin proteins. In line with a glycosylation of the skin proteins are positive reactions of B-zone material with the Schiff's base [22], [46]. After exiting the gland into the duct the spinning dope adopts the state of a nematic crystalline solution, which facilitates protein alignment and assembly [47], [48]. Within the gland lumen and throughout the duct MaSp 1 and 2 are homogeneously distributed and they seem to partially separate into different compartments as the β-sheet content increases at the peripheral regions [29]. The outer skin proteins seem not to mix with the MaSps and they might therefore be more prone to assembly, which will be enhanced by the higher shear forces near the duct walls. As a matter of fact, the skin showed the highest oriented protein chains of the whole fibre [49]. Induced by the shear flow, pH alterations and variations in specific ions combined with water elimination, polymerisation of spidroins continues into the central part of the dope [29]. Prior to the emergence of the solid fibre an extensional flow triggers conformational changes and chain alignment [34]. The final crystallisation occurs outside the spinneret by a draw down of the fibre [50]. Due to their looser attachments to the inward material, the glyco and lipid layers are likely to be added just prior to the exit of the silk material as coatings for protection and ecological purposes.

As a consequence dragline silk represents a composite material of high complexity. By altering its composition the spider is able to achieve better silk functionality and to facilitate spinning in adaptation to changing environmental conditions [51]. The primary structure of silk and the spinning conditions are likely to have an equally important impact on both micro- and supra-molecular structures of silk, which in turn define the mechanical properties of the fibre. A detailed understanding of the higher order organisation of silk will therefore be as important as full length spidroin sequences to produce synthetic fibres that biomimic spider silk's strength and toughness. So far all attempts to artificially spin a fibre of comparable quality have failed, possibly because we still have to learn to appreciate the contribution of less prominent compounds and synergic effects that are typical for composites.

## Materials and Methods

### Spiders

Livestock breeding and silking of the golden orb-weaving spider *Nephila clavipes* were done as described [26].

### Microscopic investigations

Technical details for light microscopy (LM) [28], video contrast microscopy (VCM) [31], laser scanning microscopy (LSM) [28], transmission electron microscopy (TEM) [17] and scanning electron microscopy (SEM) [52] have been outlined previously. For atomic force microscopy (AFM) ultra thin sections of dragline embedded in LR White [30] were adsorbed to glimmer. AFM measurements were performed with a NanoScope II device from Digital Instruments (Santa Barbara/California, USA) in the tapping mode [17]. Spatial (high resolution stereo) image microscopy (SIM) was performed on an Axiotech microscope (Carl Zeiss Jena, Germany).

### Silk fibre manipulations

For manipulations silk fibres were either fixed prior to or after different treatments to glass slides (LSM, TEM) as well as glass squares (5×5 mm), glimmer or aluminium blocks (SEM) by conductive silver adhesive or nail varnish. Bulk fibres were treated with different solvents in tubes, whereas solvents were overlaid onto the fixed fibres. Reagents used were the chaotropic agents lithium thiocyanate (LiSCN) at different concentrations, 8 M urea with and without Coomassie Brilliant Blue G250 staining (0.1% in PBS) and hexafluorisopropanol (Hfip, 99+% ) or a mixture of acetic acid (HAc) and hydrochloric acid (HCl) at different ratios. At various times samples were analysed by phase contrast LM using a CCD camera, by VCM or SEM.

Solubilisation experiments were carried out by incubation of approximately 1 mg of dragline silk in 1ml solution. Used solvents were 8M urea, 99% Hexafluorisopropanol (Hfip), saturated lithium chloride (LiCl), saturated lithium bromide (LiBr), saturated lithium isothiocyanate (LiSCN), 6 M guanidinium hydrochloride (GdmHCl) and 6 M guanidinium isothocyanate (GdmSCN). After 24 hrs incubation in the respective solvent the sample was centrifuged at 14 000 g to remove any particulate matter and the supernatant was dialysed against 8 M urea. After a second centrifugation step 10 µl of the supernatant were analysed by sodium dodecyl sulphate-polyacrylamide gelelectrophoresis (SDS-PAGE) in a 5–20% pre-cast gel (BioRad). The gel was either directly Coomassie stained or blotted onto nitrocellulose filters. Filters were stained with specific antibodies using the enhanced chemiluminescence (ECL) for detection.

The setup for laser ablation experiments was as detailed elsewhere [53]. The silk was exposed to a laser beam with pulse energy in the range of ∼1–5 µJ in the focal plane and a pulse repetition rate between 1 and 10 Hz. Experiments were recorded on S-VHS video film with a professional Sony (SVO-9500 MDP) videocassette recorder.

### Silk staining

Water and ether treated fibres were stained for 20 min. in a 0.2% oil red solution in 60% 2-propanol (Merck, Darmstadt). After a brief wash in 60% 2-propanol and A. bidest., fibres were analysed in transmitted light by LM.

Silk fibres were decorated with antibodies or lectins using the following work-flow: brief wash in phosphate buffered saline (PBS); saturation of unspecific binding sites for 20 minutes in PBS/2% bovine serum albumin (BSA); 3 brief washes with PBS; incubation with the primary antibody (1∶10 up to 1∶1000 dilution) or biotinylated Concavalin A (Con A; Pierce) (1 mg/ml in a 1∶100 dilution) for 2 hours; 3 brief washes in PBS; incubation with a fluorescein labelled secondary antibody (Dianova, 1∶1000) or streptavidin (1 mg/ml in a 1∶100 dilution ) coupled to gold particles (Strept-Au_40_) for 1 hour; 3 brief washes in PBS; mounting and analysis by fluorescent LM or SEM.

Decoration of ultra-thin sections with antibodies and analysis by TEM were performed as described [30].

### Separation of different silk layers

For TEM investigations silk fibres were attached to the rim of reaction tubes by adhesive tape. Fibres were first treated two times for 10 minutes in ether with gentle shaking followed by washing in water (removal of lipids). Next fibres were 10 times vigorously washed by vortexing in 0.1% Triton-X-100 (removal of glycoprotein coat). In the next step, fibres were 10 times flash frozen in liquid nitrogen. After each freezing cycle, fibres were washed in water (removal of skin layer). Cross-sections of fibres were prepared for each step and analysed by TEM.

For gel electrophoresis fibres were extracted in bulk twice with ether. The extracts were combined, the ether evaporated and the dried material suspended in Hfip (lipid fraction). Then the fibres were washed twice gently in water and mechanically sheared using a Polytron device. After that, fibres were vigorously washed 10 times by vortexing in 0.1% Triton X-100. The washes were combined and concentrated in Microcon ultracentrifugal filter devices (Millipore, nominal molecular weight limit 10 K). After washing twice with water, the retained material was suspended in Hfip (glycoprotein fraction). Next, fibres were 10 times flash frozen in liquid nitrogen, followed by quick thawing. The fibres were then incubated two times for 10 minutes in 25% lithiumbromide (LiBr) to solubilise the inner core. Particulate matter was sedimented by ultracentrifugation and suspended in Hfip (skin fraction). The solute of the supernatant, handled with filter pipet tips to avoid contamination with any floating particulate matter from the centrifugation step, was changed to Hfip by two successive centrifugation-wash cycles using Microcon ultracentrifugal devices (core fraction). All samples were then dialyzed against 8M urea. Fractions were separated on 5–20% gradient gels (BioRad), and blotted to nitrocellulose membranes. Proteins were either revealed by Ponceau S staining (Sigma-Aldrich) or reacted with biotinylated Concavalin A or silk specific antibodies. Concavalin A was detected with streptavidin and antibodies by secondary antibodies, both coupled to peroxidase. Detection was with enhanced chemiluminescence (ECL, GE Healthcare).

## References

[1] Gosline JM, Guerette PA, Ortlepp CS, Savage KN (1999). The mechanical design of spider silks: From fibroin sequence to mechanical function.. J Exp Biol.

[2] Denny MW (1980). Silks-their properties and functions. In The Mechanical Properties of Biological Materials, eds. J.F.V. Vincent, J.D. Currey.. Symp Soc Exp Biol.

[3] Hayashi CY, Shipley NH, Lewis RV (1999). Hypotheses that correlate the sequence, structure, and mechanical properties of spider silk proteins.. Int J Biol Macromol.

[4] Garb JE, Dimauro T, Vo V, Hayashi CY (2006). Silk genes support the single origin of orb webs.. Science.

[5] Motriuk-Smith D, Smith A, Hayashi CY, Lewis RV (2005). Analysis of the conserved N-terminal domains in major ampullate spider silk proteins.. Biomacromolecules.

[6] Ayoub NA, Garb JE, T RM, Collin MA, Hayashi CY (2007). Blueprint for a High-Performance Biomaterial: Full-Length Spider Dragline Silk Genes.. PLoS ONE.

[7] Hayashi CY, Blackledge TA, Lewis RV (2004). Molecular and mechanical characterization of aciniform silk: uniformity of iterated sequence modules in a novel member of the spider silk fibroin gene family.. Mol Biol Evol.

[8] Tian M, Lewis RV (2005). Molecular characterization and evolutionary study of spider tubuliform (eggcase) silk protein.. Biochemistry.

[9] Mahoney DV, Vezie DL, Eby RK, Adams WW, Kaplan D, Kaplan D, Adams WW, Farmer B, Viney C (1997). Aspects of the Morphology of dragline silk of Nephila clavipes..

[10] Thiel BL, Guess KB, Viney C (1997). Non-periodic lattice crystals in the hierarchical microstructure of spider (major ampullate) silk.. Biopolymers.

[11] Vollrath F, Knight DP (1999). Structure and function of the silk production pathway in the spider Nephila edulis.. Int J Biol Macromol.

[12] Vollrath F, Sponner A, Blackburn RS (2005). The route to synthetic silks.. Biodegradable and Sustainable Fibres.

[13] Parkhe AD, Seeley SK, Gardner K, Thompson L, Lewis RV (1997). Structural studies of spider silk proteins in the fibre.. J Mol Recognit.

[14] Porter D, Vollrath F, Shao Z (2005). Predicting the mechanical properties of spider silk as a model nanostructured polymer.. Eur Phys J E Soft Matter.

[15] van Beek JD, Hess S, Vollrath F, Meier BH (2002). The molecular structure of spider dragline silk: folding and orientation of the protein backbone.. Proc Natl Acad Sci U S A.

[16] Gould SAC, Tran KT, Spagna JC, Moore AMF, Shulman JB (1999). Short and long range order of the morphology of silk from Latrodectus hesperus (Black Widow) as characterized by atomic force microscopy..

[17] Li SF, McGhie AJ, Tang SL (1994). New internal structure of spider dragline silk revealed by atomic force microscopy.. Biophys J.

[18] Lazaris A, Arcidiacono S, Huang Y, Zhou JF, Duguay F (2002). Spider silk fibres spun from soluble recombinant silk produced in mammalian cells.. Science.

[19] Scheller J, Guhrs KH, Grosse F, Conrad U (2001). Production of spider silk proteins in tobacco and potato.. NatBiotechnol.

[20] Scheller J, Henggeler D, Viviani A, Conrad U (2004). Purification of spider silk-elastin from transgenic plants and application for human chondrocyte proliferation.. Transgenic Res.

[21] Vendrely C, Scheibel T (2007). Biotechnological production of spider-silk proteins enables new applications.. Macromol Biosci.

[22] Vollrath F, Knight DP (2001). Liquid crystalline spinning of spider silk.. Nature.

[23] Thiel BL, Guess KB, Viney C (1997). Spider major ampullate silk (drag line): smart composite processing based on imperfect crystals.. J C Microsc.

[24] Vollrath F, Madsen B, Shao Z (2001). The effect of spinning conditions on the mechanics of a spider's dragline silk.. Proc R Soc Lond B Biol Sci.

[25] Gosline J, Lillie M, Carrington E, Guerette P, Ortlepp C (2002). Elastic proteins: biological roles and mechanical properties.. Philos Trans R Soc Lond B Biol Sci.

[26] Sponner A, Schlott B, Vollrath F, Unger E, Grosse F (2005). Characterization of the Protein Components of Nephila clavipes Dragline Silk.. Biochemistry.

[27] Schulz S (2001). Composition of the silk lipids of the spider Nephila clavipes.. Lipids.

[28] Augsten K, Muehlig P, Hermann C (2000). Glycoproteins and skin-core structure in Nephila clavipes spider silk observed by light and electron microscopy.. Scanning.

[29] Sponner A, Unger E, Grosse F, Weisshart K (2005). Differential polymerization of the two main protein components of dragline silk during fibre spinning.. Nat Mater.

[30] Sponner A, Unger E, Grosse F, Weisshart K (2004). Conserved C-termini of spidroins are secreted by the major ampullate glands and retained in the silk thread.. Biomacromolecules.

[31] Vollrath F, Holtet T, Thogersen HC, Frische S (1996). Structural organization of spider silk.. The Royal Society London.

[32] Thiel BL, Kunkel DD, Viney C (1994). Physical and Chemical Microstructure of Spider Dragline: A Study by Analytical Transmission Electron Microscopy.. Biopolymers.

[33] Augsten K, Weisshart K, Sponner A, Unger E (1999). Glycoproteins and skin-core structure in Nephila clavipes spider silk observed by light- and electron microscopy.. Scanning.

[34] Knight DP, Knight MM, Vollrath F (2000). Beta transition and stress-induced phase separation in the spinning of spider dragline silk.. Int J Biol Macromol.

[35] Li SF, McGhie AJ, Tang SL (1994). Comparative study of the internal structure of Kevlar and spider silk by atomic force microscopy..

[36] Shao ZZ, Hu XW, Frische S, Vollrath F (1999). Heterogeneous morphology of Nephila edulis spider silk and its significance for mechanical properties.. Polymer.

[37] Miller LD, Putthanarat S, Eby RK, Adams WW (1999). Investigation of the nanofibrillar morphology in silk fibres by small angle X-ray scattering and atomic force microscopy..

[38] Liu Y, Shao Z, Vollrath F (2005). Relationships between supercontraction and mechanical properties of spider silk.. Nat Mater.

[39] Colgin MA, Lewis RV (1998). Spider minor ampullate silk proteins contain new repetitive sequences and highly conserved non-silk-like “spacer regions”.. Prot Sci.

[40] Gosline JM, Denny MW, DeMont EM (1984). Spider silk as rubber.. Nature.

[41] Simmons AH, Michal CA, Jelinski LW (1996). Molecular orientation and two-component nature of the crystalline fraction of spider dragline silk.. Science.

[42] Guerette PA, Ginzinger DG, Weber BH, Gosline JM (1996). Silk properties determined by gland-specific expression of a spider fibroin gene family.. Science.

[43] Huemmerich D, Scheibel T, Vollrath F, Cohen S, Gat U (2004). Novel assembly properties of recombinant spider dragline silk proteins.. Curr Biol.

[44] Huemmerich D, Helsen CW, Quedzuweit S, Oschmann J, Rudolph R (2004). Primary structure elements of spider dragline silks and their contribution to protein solubility.. Biochemistry.

[45] Dicko C, Vollrath F, Kenney JM (2004). Spider Silk Protein Refolding Is Controlled by Changing pH.. Biomacromolecules.

[46] Kovoor J (1986). L'Appareil Séricigène Dans Les Genres Nephila Leach Et Nephilengys Koch: Anatomie Microscopique, Histochimie, Affinités Avec D'Autres Araneidae.. Revue Arachnologique.

[47] Knight DP, Vollrath F (2001). Comparison of the spinning of selachian egg case ply sheets and orb web spider dragline filaments.. Biomacromolecules.

[48] Knight DP, Vollrath F, Shewry PR, Tatham AS, Bailey AJ (2003). Elastomeric Proteins: Structures, Biomechanical Properties, and Biological Roles.. Biological Liquid Crystal Elastomers.

[49] Rousseau ME, Hernandez Cruz D, West MM, Hitchcock AP, Pezolet M (2007). Nephila clavipes spider dragline silk microstructure studied by scanning transmission X-ray microscopy.. J Am Chem Soc.

[50] Riekel C, Madsen B, Knight D, Vollrath F (2000). X-ray diffraction on spider silk during controlled extrusion under a synchrotron radiation X-ray beam.. Biomacromolecules.

[51] Craig CC (2003). Spider Web and Silks. Tracing Evolution from Molecules to genes to Phenotypes..

[52] Peschke T, Augsten K, Sasama F, Gabert A (1989). Determination of gold-labelled surface receptors on single cells by X-ray microanalysis.. J Microsc.

[53] Seeger S, Monajembashi S, Hutter KJ, Futterman G, Wolfrum J (1991). Application of laser optical tweezers in immunology and molecular genetics.. Cytometry.

